# Platelet-Rich Plasma (PRP) in the Treatment of Long COVID Olfactory Disorders: A Comprehensive Review

**DOI:** 10.3390/biomedicines12040808

**Published:** 2024-04-05

**Authors:** Antonino Maniaci, Salvatore Lavalle, Edoardo Masiello, Jerome R. Lechien, Luigi Vaira, Paolo Boscolo-Rizzo, Mutali Musa, Caterina Gagliano, Marco Zeppieri

**Affiliations:** 1 Faculty of Medicine and Surgery, University of Enna “Kore”, Piazza dell’Università, 94100 Enna, EN, Italy; antonino.maniaci@unikore.it (A.M.);; 2 Research Committee of Young Otolaryngologists of International Federation of Otorhinolaryngological Societies (World Ear, Nose, and Throat Federation), 13005 Paris, France; 3 Clinical and Experimental Radiology Unit, Experimental Imaging Center, IRCCS San Raffaele Scientific Institute, Vita-Salute San Raffaele University, Via Olgettina 60, 20132 Milan, MI, Italy; 4 Department of Human Anatomy and Experimental Oncology, Faculty of Medicine, UMONS Research Institute for Health Sciences and Technology, University of Mons (UMons), 7000 Mons, Belgium; 5 Maxillofacial Surgery Operative Unit, Department of Medicine, Surgery and Pharmacy, University of Sassari, 07100 Sassari, SS, Italy; 6 Biomedical Science Department, Biomedical Science Ph.D. School, University of Sassari, 07100 Sassari, SS, Italy; 7 Department of Medical, Surgical, and Health Sciences, Section of Otolaryngology, University of Trieste, 34149 Trieste, TS, Italy; 8 Department of Optometry, University of Benin, Benin City 300238, Nigeria; 9 Eye Clinic Catania, University San Marco Hospital, Viale Carlo Azeglio Ciampi, 95121 Catania, CT, Italy; 10 Department of Ophthalmology, University Hospital of Udine, p.le S. Maria della Misericordia 15, 33100 Udine, UD, Italy

**Keywords:** olfactory dysfunction, platelet-rich plasma, anosmia, smell loss, Long COVID

## Abstract

*Background:* Long COVID has brought numerous challenges to healthcare, with olfactory dysfunction (OD) being a particularly distressing outcome for many patients. The persistent loss of smell significantly diminishes the affected individual’s quality of life. Recent attention has been drawn to the potential of platelet-rich plasma (PRP) therapy as a treatment for OD. This comprehensive review aims to evaluate the effectiveness of PRP therapy in ameliorating OD, especially when associated with long-term COVID-19. *Methods:* We executed a comprehensive search of the literature, encompassing clinical trials and observational studies that utilized PRP in treating OD limited to COVID-19. We retrieved and comprehensively discussed data such as design, participant demographics, and reported outcomes, focusing on the efficacy and safety of PRP therapy for OD in COVID-19 patients. *Results:* Our comprehensive analysis interestingly found promising perspectives for PRP in OD following COVID-19 infection. The collective data indicate that PRP therapy contributed to a significant improvement in olfactory function after COVID-19 infection. *Conclusions:* The evidence amassed suggests that PRP is a promising and safe therapeutic option for OD, including cases attributable to Long COVID-19. The observed uniform enhancement of olfactory function in patients receiving PRP highlights the necessity for well-designed, controlled trials. Such studies would help to refine treatment protocols and more definitively ascertain the efficacy of PRP in a broader, more varied patient cohort.

## 1. Introduction

Olfactory dysfunction (OD) represents a significant and pervasive impairment within the global population, affecting an estimated 20% of individuals [1]. The impact of this condition extends beyond the mere loss of smell, as it can profoundly influence quality of life, nutritional status, and personal safety [2,3]. Moreover, OD is correlated with increased morbidity and mortality rates, characterized by a spectrum of disturbances in the perception of odors [4]. Quantitative disorders affect the intensity of perceived odors, while qualitative disorders alter the character of odors or produce phantom olfactory perceptions. Qualitative changes, such as parosmia, typically manifest with alterations perceived negatively, and are infrequent in isolation, commonly co-occurring with quantitative impairments [5]. From an anatomical perspective, OD can be divided into three primary categories based on the lesion’s location: conductive, sensorineural, and central [6]. However, this classification can be overly simplistic, and may not fully encapsulate the complex pathophysiology of the condition, as these categories often intersect. 

Olfactory dysfunction is a multifaceted condition with roots extending across various etiologies [7]. Infectious agents, notably including those responsible for COVID-19, can leave a lasting impact on our sense of smell, a phenomenon known as post-infectious olfactory dysfunction [8]. Meanwhile, inflammatory sinonasal diseases often impede olfactory processes, just as head traumas can physically disrupt olfactory pathways [9]. Neurological disorders introduce another dimension, as degenerative changes within the brain can impair olfactory functionality. External agents, such as certain drugs or toxins, can also diminish our olfactory acuity [10,11]. Moreover, some individuals are born with a compromised sense of smell, while others encounter this decline as a natural part of aging [12,13]. Lastly, medical interventions and unexplained causes can lead to olfactory dysfunction, further complicating the diagnostic and treatment landscape [7]. Each etiology presents its unique challenges and underscores the need for a customized approach to understanding and addressing olfactory dysfunction. 

The COVID-19 pandemic has highlighted the prevalence of the related OD, with a significant proportion of patients reporting anosmia as a primary symptom, often coupled with gustatory dysfunction [14]. Anosmia has been recognized as a robust predictive marker for COVID-19, particularly in asymptomatic carriers, emphasizing the need for effective diagnostic and treatment strategies [15,16]. Despite the prevalence and impact of OD, therapeutic options remain limited and largely ineffective in the long term [17]. This is primarily due to a shortage of robust evidence in the literature, a consequence of insufficient research funding, inadequate participant numbers, and methodological diversity that hampers the generalizability of study outcomes. The urgency imposed by the pandemic has, however, catalyzed research efforts and funding toward developing treatments for OD [18]. Current treatment recommendations for OD include the administration of systemic and intranasal corticosteroids, particularly for inflammatory conditions such as chronic rhinosinusitis (CRS) and severe allergic rhinitis [19]. For intranasal corticosteroid delivery, mechanisms that target the olfactory cleft are favored. 

Olfactory training has also been suggested for various etiologies, acknowledging that further evaluation is required for its efficacy in inflammatory and neurodegenerative conditions [20]. Functional endoscopic sinus surgery is endorsed for CRS-induced olfactory loss, adhering to established guidelines. In cases of severe CRS with nasal polyposis, biological treatments like dupilumab have shown improvements in OD [21,22]. Amidst these options, platelet-rich plasma (PRP) has emerged as a novel and promising therapeutic avenue for OD [23,24]. PRP is an autologous concentration of platelets in a small volume of plasma, boasting anti-inflammatory and pro-regenerative properties. It contains growth factors such as TGF-beta, EGF, VEGF, NGF, and IGF, which are instrumental in promoting tissue healing and regeneration [25,26]. These capabilities of PRP have been harnessed in various clinical and surgical contexts since the 1970s [27,28,29,30,31] and are now being explored for their potential in olfactory tissue repair and neuroregeneration [32,33]. Animal studies have shown that growth factors and stem cells can effectively treat anosmia and regenerate olfactory neuroepithelium, positioning PRP as a promising candidate for neuroregenerative therapies [34,35]. 

In clinical practice, PRP has been gaining traction within otolaryngology for its diverse applications, ranging from enhancing wound healing post-surgery to treating sensorineural hearing loss, and even as a treatment for qualitative OD [36,37,38,39,40]. While the literature contains systematic reviews on the use of PRP in the context of COVID-19-related OD [41], comprehensive reviews evaluating PRP’s efficacy across other etiologies of OD are lacking. This comprehensive review aimed to fill this gap by revisiting the current literature on PRP use in olfactory dysfunction caused by CRS, trauma, anesthetic exposure, or viral infections, including COVID-19. In addition, we seek to elucidate the reality of PRP as a treatment for olfactory dysfunction after COVID-19 infection. 

## 2. Materials and Methods

### Literature Research and Study Design

**Figure 1:**
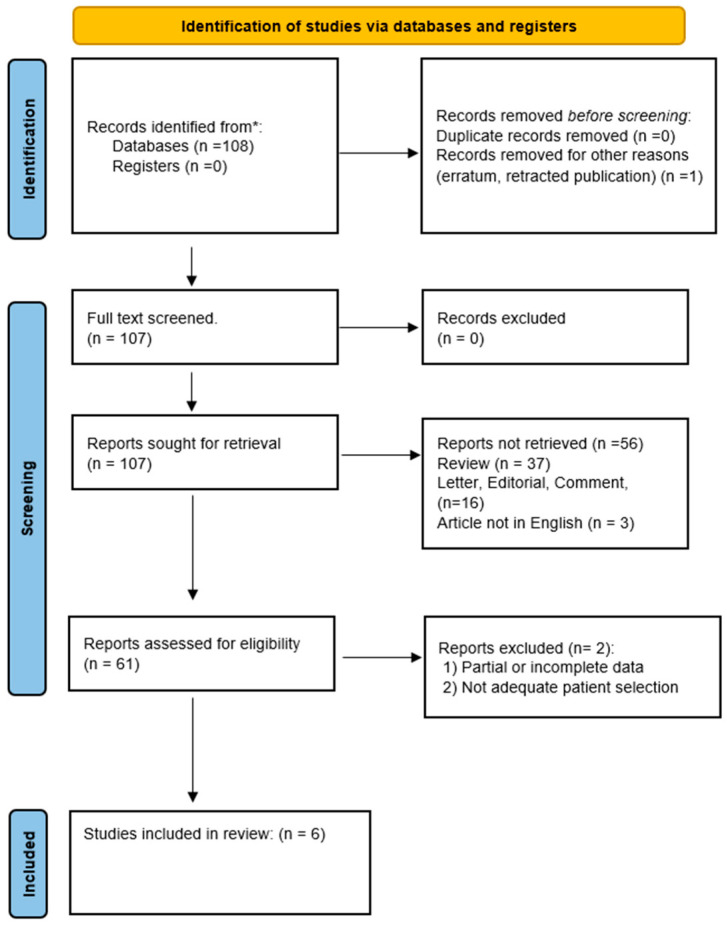
PRISMA flow diagram describing the literature research protocol. * PubMed, SCOPUS, EMBASE, Web of Science, and Cochrane.

This literature research was conducted to examine the effects of PRP in treating OD in adults. A comprehensive literature search was performed to identify studies up to November 2023 from databases including PubMed, SCOPUS, EMBASE, Web of Science, and Cochrane. Search terms were tailored to each database and included a combination of the following: “platelet-rich plasma”, “PRP”, “Olfactory dysfunction”, “anosmia”, “hyposmia”, “parosmia”, “COVID-19 olfactory dysfunction”, and “Functional endoscopic sinus surgery OR FESS.” Cross-referencing ensured thorough coverage (Figure 1). 

All studies involved adult patients treated with PRP for OD due to COVID-19. The interventions considered were in-office and peri-operative PRP injections into the nasal fossae, with comparisons between pre and post-treatment outcomes. Outcomes were evaluated using objective tests (e.g., TDI, STC, SIC), self-reported tests (e.g., VAS for parosmia and ODQ), and recorded side effects. The analysis involved a comprehensive synthesis, reporting outcomes after PRP treatment with a control or placebo group during follow-ups when possible.

## 3. Results

### 3.1. OD in Long-COVID-19

Long COVID-19 is characterized by a diverse range of persistent symptoms, including fatigue, brain fog, and various forms of organ dysfunction [8,14]. One of the notable sequelae of Long COVID-19 is olfactory dysfunction (OD), which can last for months after the initial SARS-CoV-2 infection [15,16,17]. The precise mechanisms underlying the development of OD in Long COVID are not yet fully understood, but several hypotheses have been proposed [18,41]. Persistent viral infection or reactivation within the olfactory system may lead to chronic inflammation and damage to the olfactory neurons or supporting cells [42,43]. Alternatively, the initial infection may trigger maladaptive immune responses, such as autoantibody production targeting olfactory structures, contributing to persistent OD [44,45]. The neurotropic nature of SARS-CoV-2 may also result in direct or indirect effects on the olfactory neural pathways, impacting olfactory perception and processing [46,47].

Also, some Long COVID patients have observed microvascular injury and thrombotic events affecting the olfactory bulb or central olfactory regions, potentially disrupting normal olfactory function [48,49]. Elucidating the complex pathophysiology of OD in Long COVID is crucial for developing targeted interventions to alleviate this debilitating symptom experienced by many affected individuals. 

Therapies that target the mechanisms of chronic viral infection, dysregulated immunological responses, and microvascular dysfunction that underlie OD in Long COVID may prove advantageous [37,50]. In various clinical contexts, platelet-rich plasma (PRP), a concentrated source of autologous growth factors and cytokines produced from the patient’s own blood, has demonstrated promise in promoting tissue repair and regeneration [34,41]. PRP therapy may be beneficial in the context of OD linked to Long COVID through several ways. PRP’s growth factors and anti-inflammatory mediators may be able to mitigate the negative effects of ongoing viral infection or autoimmune reactions on the olfactory system, as well as lessen chronic inflammation. Furthermore, PRP’s angiogenic and regenerative qualities might aid in re-establishing the olfactory bulb’s microvascular integrity and perfusion [25,35].

### 3.2. Definition, Composition of PRP, and Molecular Mechanisms

PRP is defined as a volume of the plasma fraction of autologous blood having a platelet concentration above baseline [22]. It is produced by centrifuging the patient’s own blood to yield a concentrated suspension of platelets in a small volume of plasma. When exploring the therapeutic applications of PRP, the distinction between autologous and heterologous (also known as allogeneic) sources is a critical factor to consider [23,24,25]. Autologous PRP, derived from the patient’s blood, stands in contrast to heterologous PRP, obtained from a donor. Autologous PRP has garnered favor in the medical community due to its inherent compatibility with the patient’s own immune system [28]. 

The process involves collecting and centrifugation the patient’s blood to concentrate the platelets within the plasma, which is then reintroduced into the patient’s body at the site of injury or tissue damage [26]. This closed-loop system, wherein the patient’s own biological material is used, significantly reduces the risk of immunogenic reactions and the potential for disease transmission. The body naturally recognizes the reintroduced cells and proteins as part of itself, virtually eliminating the risk of rejection or allergic reactions that can complicate recovery. On the other hand, heterologous PRP comes with a set of challenges that cannot be overlooked [29,30,31]. Derived from a donor’s blood, heterologous PRP introduces foreign proteins and cells into the patient’s body, raising concerns about immunocompatibility. 

The immune system may identify the infused heterologous platelets and plasma proteins as foreign elements, leading to an immune response ranging from mild inconvenience to severe complication [50]. Additionally, despite rigorous screening and testing of blood products, the use of donor-derived PRP carries a residual risk of transmitting infectious diseases, such as HIV or hepatitis, from the donor to the recipient [51]. Moreover, the potential for rejection and allergic reactions is more pronounced with heterologous PRP [52]. Such responses can hinder the healing process and reduce the therapeutic benefits PRP intends to provide. Consequently, the medical community strongly prefers autologous PRP, given its superior safety profile and alignment with the principles of personalized medicine. The choice between autologous and heterologous PRP is often clear-cut, with the former being the preferred method for reducing complications and enhancing the body’s natural regenerative capacity. Clinicians and patients tend to favor autologous PRP for its patient-specific approach to treatment, which minimizes risks and offers a tailored therapy that capitalizes on the patient’s own healing mechanisms [34,37]. 

The PRP that is utilized in various therapeutic interventions boasts a composition teeming with platelets, far exceeding the concentration found within normal blood. These platelets are not just components that respond swiftly to injury, but also factories of growth factors and cytokines crucial for healing [41]. In typical PRP preparations, the goal is to achieve a platelet concentration 3–5 times that of baseline blood levels, translating into a potent cocktail of biologically active proteins [34]. The array of proteins released from these platelets includes Platelet-Derived Growth Factor (PDGF), which drives cell replication and the formation of new skin and blood vessels; Transforming Growth Factor Beta (TGF-β), which is essential in healing bone and soft tissues; Vascular Endothelial Growth Factor (VEGF), an important molecule in blood vessel formation; Epidermal Growth Factor (EGF), which plays a role in cell growth and the production of collagen; and Fibroblast Growth Factor (FGF), which aids in wound repair. 

PRP contains a slew of interleukins, chemokines, and other cytokines that regulate inflammation and cell communication. The presence of proteins such as fibrin, fibronectin, and vitronectin further enhances the healing properties of PRP [31]. Fibrin creates a matrix that facilitates cell migration and tissue regeneration. At the same time, fibronectin and vitronectin are adhesion molecules that promote cellular attachment and spreading, all of which are essential in the wound-healing cascade. PRP operates through a series of complex molecular mechanisms, starting with forming a fibrin mesh during hemostasis, which not only halts bleeding, but also provides a framework for cells to move across [27,28]. The growth factors and cytokines within PRP modulate the inflammatory response, which is crucial for preparing the wound bed for new tissue. Cellular proliferation is stimulated by PDGF and EGF, which multiply the cells needed for repair. 

Angiogenesis follows, with VEGF and FGF inducing new blood vessel formation, ensuring the delivery of essential nutrients and waste disposal [29]. TGF-β oversees the remodeling phase, where collagen synthesis by fibroblasts fortifies tissue strength and integrity. These growth factors also serve as chemotactic signals, recruiting macrophages and stem cells to the injury site and emphasizing PRP’s role in tissue repair and regeneration [53,54,55]. The fibrin matrix’s scaffolding effect and the modulation of inflammation by PRP contribute to reduced healing time and improved recovery outcomes [56]. In cases like olfactory disorders, where the olfactory epithelium’s capacity to regenerate is crucial, PRP might offer significant therapeutic benefits. The growth factors in PRP could potentially enhance the natural regenerative process of the olfactory epithelium, encouraging the differentiation of basal stem cells into functional olfactory neurons, thus aiding in the restoration of smell.

### 3.3. PRP Protocol Preparation

The literature to date presents 6 papers dealing with the results of PRP after COVID-19 infection. All these studies included individuals treated with PRP for olfactory dysfunction post-COVID-19 infection (Table 1). 

The studies found in the literature ranged in publication date from January 2021 to November 2023. The participants were predominantly adults with a mean age of 45 years, spanning from 18 to 65 years. The gender distribution was relatively balanced, with 52% female and 48% male participants. All individuals had a confirmed history of COVID-19 infection and exhibited varying degrees of olfactory dysfunction for at least three months post-recovery from the infection. PRP preparation and application procedures were consistent across studies, with slight variations in the volume of blood drawn and centrifugation specifics. 

Different PRP injection techniques are described in the research; some use a single injection, while others use repeated injections spaced out over a shorter period of time [54,55,56,57,58,59]. Most injections were administered at outpatient facilities or hospital based setting, and the frequency of treatment sessions ranged from a single application to multiple injections over a period of up to six weeks. Yan and colleagues specifically used three PRP intranasal injections spaced two weeks apart into the olfactory cleft [55]. In comparison, one PRP injection into the olfactory cleft was used by Steffens et al. [56] and Lechien et al. [54,58]. In order to achieve a compromise, Abo El Naga et al. gave the olfactory cleft three PRP injections spaced three weeks apart [59]. Lastly, a single injection of 1 mL of PRP into the olfactory cleft region was employed by Evman et al. [59]. Abo El Naga et al.’s injection protocol was the longest ever documented, lasting a total of six weeks (three doses spaced three weeks apart) [57]. The absence of a protracted multi-injection PRP strategy in the literature review indicates that more research is needed to determine the best way to administer PRP and whether it is beneficial in treating COVID-19-related olfactory impairment. To find out if a prolonged therapy plan involving multiple PRP injections could help individuals with COVID-19 infection-related persistent olfactory impairment or improve their results, more research may be required.

**Figure 2:**
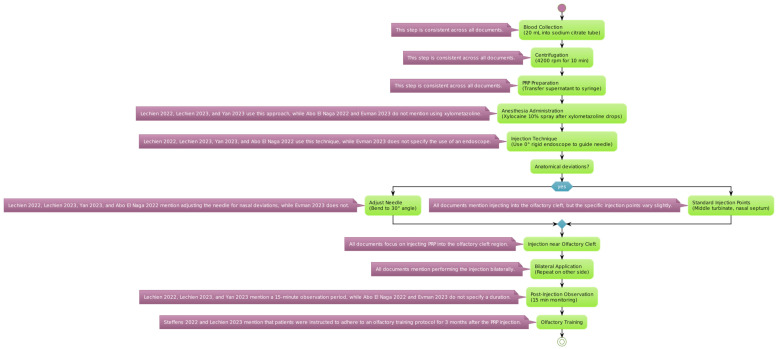
PRP protocol for extraction and injection. Variations in platelet-rich plasma preparation and administration are detailed for each specific step [54,55,56,57,58,59].

The reviewed studies employed a standardized protocol for the extraction and application of PRP, starting with an initial blood draw of 20 mL, with the sample being placed into a tube containing sodium citrate as an anticoagulant (Figure 2).

The blood sample was centrifugated at a speed of 4200 revolutions per minute (rpm) for 10 min. This process separates the platelet-rich plasma from other blood components [53]. The plasma layer with a high concentration of platelets (the supernatant) was then carefully transferred into a 10 mL syringe for administration. 

Anesthesia Administration: Before the injection, local anesthesia was achieved using a 10% Xylocaine spray. This was applied 2 min after instilling xylometazoline hydrochloride drops into the nasal passages to prepare the area and reduce discomfort during the procedure. Consequently, a 0° rigid endoscope was used to accurately guide the needle into position. The needle may be adjusted to a 30° angle to optimize the access to the targeted nasal anatomy. 

It is evident from reading through the several publications on the application of PRP in carefully chosen and strategic areas that the investigators took distinct techniques with regard to the particular anatomical sites. Notably, only the Lechien et al. 2022 trial offered comprehensive details regarding the injection sites [54]. The authors reported that they injected 0.2–0.5 mL of PRP into many locations in the middle turbinate as well as the nasal septum in relation to the middle turbinate head. The other investigations, in comparison, adopted a more generic strategy, merely reporting that the PRP injections were given into the “olfactory cleft” or “olfactory region” without going into detail about the specific anatomical landmarks that were used as guidance for the injection. It is difficult to directly compare the techniques and possibly determine any benefits or drawbacks to targeting particular nasal structures, like the middle turbinate, as opposed to a more generalized olfactory cleft approach, because of the disparity in reporting the injection sites across the various studies. Thus, when assessing and combining the results from the literature, the variations in reporting the anatomical targets should be taken into account.

In patients exhibiting anatomical deviations, the PRP injections were administered as close as possible to the olfactory cleft to ensure efficacy. The same injection process was replicated on the opposite side of the nasal cavity to provide a comprehensive treatment approach. Following the injection, patients were monitored for a duration of 15 min to watch for any immediate adverse effects before being discharged. 

#### Differences in Preparation and Administration

The volume of whole blood collected, the anticoagulant employed, the centrifugation settings, and the final PRP injection volume were the main variations between the trials. The final therapeutic qualities of PRP, including its growth factor content and platelet concentration, may be affected by these differences in PRP preparation and administration. In order to obtain the platelet concentrate, Abo El Naga et al. [57] provide a detailed methodology for PRP preparation that involves a “soft spin” centrifugation at 800 rpm for 10 min, followed by a “hard spin” at 2000 rpm. An amount of 1–2 mL of PRP were used for the intranasal injections. Lechien et al., in contrast, did not include a detailed description of the PRP preparation technique in their 2022 paper [54], instead referring to the authors’ earlier work’s methodologies. They injected one milliliter of PRP bilaterally into the olfactory cleft in this initial uncontrolled research. The authors of the larger controlled trial, which was published in 2023 [58], included more details about the PRP preparation, indicating that it was done in accordance with previous publications’ methods. Yan et al. [55] simply said that the PRP was prepared in accordance with previously known protocols, without going into great detail about the preparation process. One milliliter of PRP was injected into the olfactory cleft. A less complicated method was employed by Evman et al. [59], who injected a single dosage of 1 mL of PRP into the olfactory cleft region without mentioning the centrifugation parameters. Steffens et al. [56] said they utilized a similar preparatory technique by citing Yan et al.’s [55] methodology for their PRP injections.

### 3.4. Efficacy of PRP Treatment and Comparative Analysis

**Figure 3:**
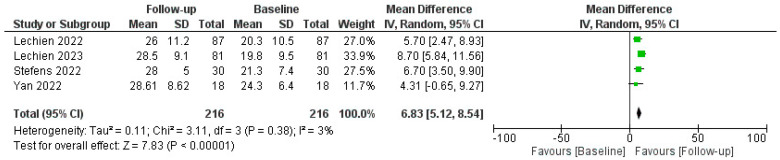
Forest plot describing pooled TDI outcomes after PRP injection. Abbreviations: TDI, Threshold, Discrimination, Identification; PRP, platelet-rich plasma. Green squares represent each mean difference of study described while rombus the overal mean differences of all the studies included.

The efficacy of PRP in restoring olfactory function was measured using various tools, including the Threshold, Discrimination, and Identification (TDI) score, Sniffin’ Sticks test (SST), and self-reported questionnaires. The pooled data revealed a statistically significant improvement in olfactory function in patients post-PRP treatment, as reported by mean TDI score, SST, and patient-reported outcomes improvements; a comprehensive analysis of the administration of Platelet-Rich Plasma (PRP) injections for COVID-19-related olfactory dysfunction (C19OD) revealed promising outcomes across multiple studies, as described in Figure 3. 

Lechien J.R. et al. conducted two studies [54]. The first was without a control group, demonstrating significant improvement in both ODQ and TDI scores post-PRP injection despite some temporary side effects like epistaxis and parosmia related to xylocaine spray. Their follow-up-controlled study also revealed significant improvements in olfactory function in PRP-treated patients compared to untreated controls (*p* = 0.001). CHH et al. [55] also reported improved olfaction (TDI increase of 3.67 points) in PRP recipients, with notable enhancements in smell discrimination but not in identification or subjective scores, and again without adverse events. Steffens Y. et al. [56] implemented PRP injections in 56 patients, significantly improving mean TDI scores by 6.7 points (*p* < 0.001) and self-assessed olfactory function (1.8 on a mild-to-moderate scale) compared to controls who received only olfactory training. No adverse effects were observed. 

Evman M. et al. [59] investigated PRP treatment in patients with long-term C19OD unresponsive to other therapies. The PRP group exhibited significant enhancement in both smell threshold and identification capacities (*p* = 0.037 and *p* < 0.001, respectively) compared to controls, with no reported adverse events. Lastly, El Naga H. et al. [57] focused on patients with post-COVID olfactory parosmia, administering three PRP injections at three-week intervals. The PRP group saw a significant reduction in parosmia severity compared to controls (*p* = 0.002). Collectively, these studies underscored the potential of PRP injections as a safe and effective intervention for persistent olfactory disorders following COVID-19, with significant improvements in objective and subjective olfactory measures.

### 3.5. Factors Influencing PRP Treatment

The efficacy of PRP therapy as a treatment for olfactory disorders associated with COVID-19 is influenced by an intricate interplay of factors [41,54,55]. The unique characteristics and demographics of each patient—including age, sex, overall health status, and the presence of underlying medical conditions—can significantly affect how one might respond to PRP therapy [56]. For instance, younger patients with robust healing capabilities may experience quicker and more complete recovery than older individuals or those with comorbidities that may impede the regenerative process [57,58,59,60]. 

Timing is another critical aspect. Initiating PRP treatment too early or too late during olfactory dysfunction could influence its effectiveness [61,62]. Similarly, the duration of treatment needs to be carefully calibrated; too short a course may be insufficient for recovery, while too long a course could lead to diminishing returns [63]. The specific composition and preparation methods of the PRP are also vital. 

The concentration of platelets, which are the source of growth factors essential for tissue regeneration, must be optimized. The exact methodology used in extracting and preparing PRP, such as the speed and duration of centrifugation, can affect the quality of the final product [53]. Moreover, the method of activating the platelets to release these growth factors can have implications for treatment efficacy. 

PRP therapy does not exist in a vacuum; concomitant treatments and interventions that a patient undergo for COVID-19 or its after-effects could influence the healing process [23]. Medications, other therapies, or nutritional supplements might interact with PRP in ways that can either enhance or detract from its regenerative capacity [30]. Understanding and navigating these factors is critical for clinicians when developing a PRP treatment plan for COVID-19-related olfactory disorders. By considering each patient’s individual needs and circumstances, optimizing PRP composition and preparation, and carefully coordinating additional treatments, healthcare providers can improve the chances of successful olfactory recovery for their patients.

### 3.6. PRP Treatment Duration

Studies assessing the use of PRP for COVID-19-related olfactory impairment have reported varying lengths of follow-up and treatment durations. The PRP group in the El Naga et al. study had a longer treatment plan, consisting of three PRP injections every three weeks for a total of nine weeks of treatment [57]. By comparison, the control group in that study only followed the pre-study treatment program for six weeks. The trial conducted by Evman et al. employed a somewhat abbreviated treatment duration, wherein all patients were first treated for one month with nasal treatments and olfactory rehabilitation [59]. Patients who did not respond to this first regimen were then randomized to get a single PRP injection or to continue receiving control treatment. All patients were monitored for an extra month, making the entire duration of the study two months. 

In contrast, the Lechien et al. investigations used a single PRP injection and assessed results two months (2022) [54] and ten weeks (2023) [58] after the injection. The 2022 study found that following a single PRP injection, the mean time to increase olfactory perception noticeably was 3.6 weeks. Ultimately, a compromise was offered by the Yan et al. trial [55], which involved the administration of three PRP or placebo injections spaced two weeks apart for a total treatment period of four weeks. The PRP treatment durations in these trials have varied from one injection to three injections spaced out across four to nine weeks, with follow-up periods lasting between one and two months or longer following the last injection. This difference in treatment procedures highlights the need for more research to determine the best PRP regimen—including the ideal number of injections and timing of follow-up assessments—for COVID-19-related olfactory impairment. 

### 3.7. PRP Safety and Tolerability for COVID-19 OD

PRP treatment was reported to be well-tolerated, with minimal adverse effects. In the landscape of studies investigating the side effects of PRP injections for COVID-19-related olfactory dysfunction, we find a variance in the reported outcomes, although the overarching narrative hints at a favorable safety profile. No adverse events related to PRP injections were reported across the studies. Steffens Y. et al. and Yan C.H. et al. [55,56] presented a scenario where PRP injections did not elicit adverse reactions, a finding that bodes well for patient tolerance. 

Conversely, in the studies by Lechien J.R. et al. [58], a spectrum of side effects emerged. Their preliminary research revealed moderate, but not insignificant, adverse responses, including transient epistaxis, which may have been expected considering the treatment’s nasal delivery. The xylocaine spray, which is used for its anesthetic qualities, has been associated with temporary cases of parosmia. Additionally, a small percentage had vasovagal episodes, which serve as a reminder of the body’s occasionally erratic reaction to medical procedures. Even though it was subjective, patients’ pain ratings ranged from mild to moderate. However, Lechien J.R. et al. did not emphasize side effects in their follow-up study, suggesting that such events may not have been a substantial concern or were managed effectively in the controlled setting [60]. 

Similarly, Evman M. et al. documented a clean slate concerning adverse reactions, further supporting the notion that PRP injections can be a tolerable treatment option [59]. While some minor and primarily transient side effects have been observed in certain studies, the general consensus points to PRP therapy as a safe procedure for those suffering from olfactory impairments post-COVID-19 infection [61]. However, it is important to remember that the lack of reported side effects in some studies does not preclude their occurrence, and that patient experiences can be as diverse as the studies themselves. Despite the promising results, there were limitations noted in the data. Several studies lacked long-term follow-ups, which limited the ability to assess the persistence of PRP effects on olfactory recovery. 

## 4. Discussion

The COVID-19 pandemic has spotlighted the longstanding and pervasive challenge of managing OD [62]. Quantitative smell deficits and qualitative distortions such as parosmia have always posed difficulties for otolaryngologists, given the complexity of olfactory pathways and limited therapeutic options [63]. This comprehensive literature review demonstrates that PRP injections represent a promising new treatment approach for OD of varied post-viral etiology [64]. The recruitment of patients with persistent, treatment-refractory olfactory dysfunction after COVID-19—a patient population with an evident unmet need for efficacious interventions—was a common theme among these trials presents in the literature. 

The changing knowledge of the natural history and persistence of this COVID-19 issue is probably reflected in the differences in the precise duration and severity criteria used to identify olfactory impairment. However, the continuous enrolment of patients with persistent, challenging olfactory impairment highlights the potential clinical utility of PRP as a novel therapeutic strategy in this setting. Participant “persistent olfactory dysfunction” was the only need in El Naga et al.’s study [57]; neither the dysfunction’s length nor severity were specified. On the other hand, individuals who exhibited olfactory impairment that remained unresponsive after one month of initial treatment and who had contracted acute COVID-19 infection were included in the Evman et al. study [59]. Patients with notably prolonged olfactory impairment were included in the Lechien et al. investigations [54,58]; their mean durations before PRP therapy were 15.7 ± 7.5 months in the 2022 study and 14.8 ± 7.3 months in the 2023 research. Similarly, patients with olfactory impairment continuing for at least two months following COVID-19 were included in the Steffens et al. trial. The Yan et al. study adopted a more targeted strategy, concentrating on individuals with objectively determined olfactory impairment (UPSIT ≤ 33) that remained 6–12 months after COVID-19 infection and who had previously tried other conventional therapies, such as topical nasal sprays and olfactory training [55]. 

Across the analyzed original studies, patients treated with intranasal PRP injections exhibited statistically significant improvements in objective olfactory test scores and subjective smell function compared to control groups [55,56,65]. The compiled evidence indicates that PRP facilitates damaged olfactory neuroepithelium regeneration by releasing endogenous growth factors and stem cells [66]. While the exact mechanisms are still being elucidated, PRP appears to stimulate the neurogenesis and recovery of olfactory neurons and sustentacular cells while restoring nasal mucosal integrity [67]. 

Critically, PRP seems to be a well-tolerated intervention for OD patients based on the safety data reported [56]. Since PRP harnesses cytokines and bioactive factors from the ‘patient’s own platelets, risks of rejection or infection are minimized compared to exogenous compounds [68]. The reviewed articles did not report any major adverse events, just mild side effects like temporary nasal discomfort, epistaxis, or parosmia in a subset of patients, and no systemic effects were noted [69,70]. Injecting PRP directly into the nasal fossae allows targeted delivery and avoids the indefinite systemic corticosteroid usage associated with local and systemic side effects [71]. The in-office procedure, requiring only local anesthesia, also enhances accessibility and cost-effectiveness. However, some key limitations temper the results and reveal gaps necessitating further research. A lack of standardized administration protocols was noted, including optimal PRP dose, injection frequency, technique, and duration of treatment. Most studies provided only short-term follow-ups of 1–3 months, and lacked assessments of long-term durability beyond 6–12 months [41,56,63]. 

The variability in efficacy measures also prevented quantitative meta-analysis. While TDI scores were commonly reported, other validated psychophysical tests like ‘Sniffin’ Sticks were inconsistently implemented. Patient-reported outcome measures were also heterogeneous when included, utilizing non-validated surveys. Moreover, the limited number of available studies restricts the generalizability of findings to diverse OD populations. Additional factors like the ideal timing of interventions for maximal benefit and the expected length of olfactory improvements remain unknown [72]. Elucidating these open questions will require rigorous randomized controlled trials on larger cohorts over extended periods. Standardizing PRP protocols and consistently administering psychophysical testing at predefined time points will allow for meaningful quantitative analyses. Detailed baseline profiling of OD subtypes and olfactory nerve status using imaging and olfactometry can help identify the best candidates for PRP therapy. Comparing outcomes across interventions like corticosteroids and olfactory training will also inform implementation guidelines. 

A thorough assessment of adverse effects and stratification by variables such as dosage and injection frequency can optimize safety. Such methodical clinical investigation will help establish if PRP injections can become a first-line therapy for the diverse landscape of OD patients. As the medical community grapples with the long-term effects of COVID-19, particularly olfactory disorders, future directions and research priorities are being shaped to enhance the therapeutic potential of platelet-rich plasma (PRP) treatment [7,15,18]. The optimization of PRP preparation and administration protocols is a critical area of focus. This involves a concerted effort to fine-tune the variables that contribute to the efficacy of PRP, such as the density of platelet concentration, the purity of the preparation, and the activation technique employed to induce the release of growth factors [26,27]. Standardizing these protocols will be paramount to ensure that patients everywhere receive high-quality and consistent treatment. Simultaneously, research into identifying biomarkers and predictors of treatment response will become increasingly important [24].

The discovery of specific indicators—whether they be proteins, genes, or other molecular signatures—that can predict how well a patient will respond to PRP therapy could revolutionize patient selection and treatment customization. Personalized medicine, guided by these biomarkers, might enable more precise therapy targeting, thus improving outcomes and reducing unnecessary treatments. Furthermore, large-scale multicenter clinical trials with long-term follow-ups are essential to establish a robust evidence base for using PRP in treating post-COVID-19 olfactory disorders. These trials would provide invaluable data on the effectiveness, safety, and durability of PRP therapy. They would also allow for exploring different dosing regimens, treatment intervals, and follow-up durations to understand the long-term implications of PRP treatment better. In addition to these trials, research should explore PRP in combination with other therapeutic modalities. This could include studying the effects of combining PRP with olfactory training, anti-inflammatory drugs, or even novel pharmaceutical agents designed to promote nerve regeneration. Investigating how PRP therapy can be integrated into a multimodal treatment approach may yield synergistic effects, leading to more comprehensive care strategies.

As our understanding of the pathophysiology of COVID-19-related olfactory dysfunction deepens, research priorities will also examine the fundamental mechanisms by which PRP may affect the olfactory pathways at the cellular and molecular levels. This deeper insight will inform future therapeutic innovations, and may uncover new avenues for applying PRP therapy beyond olfactory disorders, potentially benefiting a wider range of conditions resulting from viral illnesses.

## 5. Conclusions/Future Directions

Emerging promise was found for platelet-rich plasma as a therapeutic modality for diverse causes of olfactory dysfunction. The current evidence demonstrates that PRP injections can significantly improve smell function based on objective testing and patient-reported measures. PRP appears to facilitate olfactory neuroepithelium regeneration by releasing endogenous growth factors. The therapy is well-tolerated with a favorable safety profile. However, limitations like small sample sizes, lack of standardized protocols, and short-term follow-ups temper the results. High-quality randomized controlled trials with extended monitoring are needed to corroborate preliminary findings, optimize protocols, and establish definitively if PRP represents a viable first-line treatment for smell disorders. Addressing these evidence gaps through rigorous investigation will actualize the potential of this autologous regenerative therapy amidst the evolving paradigm for olfactory dysfunction management. Although promising, more research is imperative to confirm PRP’s efficacy and durability across larger populations.

## Figures and Tables

**Table 1:** Main studies included demographic data and treatment outcomes.

Reference	Location	Study Design	Group Size		Mean Age		Gender Ratio (M/F		OD Identified	OD Duration		Olfactory Outcomes	Adverse Effects
			*PRP*	*No PRP*	*PRP*	*No PRP*	*PRP*	*No PRP*		*PRP*	*No PRP*		
Steffens et al., 2022 [56]	Belgium	Prospective	30	26	39 ± 12	44 ± 11	14/16	6/20	Post-COVID-19 chronic olfactory dysfunction: 56 patients	10.8 months	9.7 months	The PRP group’s mean self-assessment of improvement in smell function was 1.8 (mild-to-moderate), significantly higher than the control group’s score of 0.3 (*p* < 0.001).	NA
El Naga et al., 2022 [57]	Egypt	Pilot study	30	30	28.9	30.07	11/19	9/21	Post-COVID-19 parosmia: 60 patients	>3.0 months	-	The VAS for parosmia showed a substantial improvement in the control group (*p* = 0.00148) and a highly significant improvement in the case group (*p* < 0.00001). Regarding the extent of improvement, there was a significant difference between the two groups that favored the case group (*p* = 0.002).	NA
Yan et al., 2022 [55]	United States	RCT	18	12	44.6	43.4	9/9	6/6	Post-COVID-19 olfactory dysfunction: 30 patients	8.6 months	8.9 months	Both the PRP and placebo groups demonstrated a substantial improvement in VAS scores at one and three months compared to baseline when evaluating subjective changes in smell function. Nevertheless, there was no discernible difference between the PRP and placebo groups in terms of the subjective olfaction scores using VAS at one or three months.	NA
Lechien et al., 2022 [54]	Belgium	Prospective	87	-	41.6 ± 14.6	-	25/62	-	Post-COVID-19 anosmia: 30 patients; post-COVID-19 hyposmia: 40 patients; post-COVID-19 parosmia: 17 patients	15.7 months	-	Twenty patients (54%) and nine patients (24%) reported significant improvement in anosmia/hyposmia or parosmia, respectively, while eight patients (22%) did not report any subjective improvement in olfactory impairment. Based on the patients’ experiences, olfaction significantly improved after a mean of 3.6 ± 1.9 weeks.	Transient epistaxis, temporary cases of parosmia due to the xylocaine spray, a small percentage had vasovagal episodes, and pain ranged from mild to moderate.
Lechien et al., 2023 [58]	Belgium; Italy; France	Multicenter controlled study	81	78	43.5 ± 13.4	47.0 ± 11.1	20/61	26/52	Post-COVID-19 anosmia: 55 patients; post-COVID-19 hyposmia: 79 patients; post-COVID-19 parosmia: 25 patients	15.7 months	11.0 months	An average improvement in subjective smell lasting 3.4 ± 1.9 weeks was noted by 85% of PRP patients. After ten weeks, the PRP group’s parosmia, life quality, TDI, and overall and sub-ODQ scores were significantly decreased. The control group experienced a significant rise in discrimination, identification, and overall TDI scores, but the ODQ score did not change. The PRP group outperformed the controls regarding 10-week TDI and ODQ scores.	NA
Evman et al., 2023 [59]	Turkey	RCT	12	13	31.8 ± 6.9	33.5 ± 11.1	6/6	6/7	Post-COVID-19 olfactory dysfunction: 25 patients	>12.0 months	-	The mean score for the smell identification test increased significantly in the PRP group, going from 11.42 (SD 1.17) to 15.17 (SD 0.39), and the mean score for the smell detection threshold increased correspondingly, going from 5.63 (SD 0.68) to 6.46 (SD 0.45). Conversely, the control group experienced a slight rise in the mean smell detection threshold score, going from 5.69 (SD 0.66) to 5.77 (SD 0.70), and a lesser increase in the mean smell identification test score, from 11.20 (SD 1.12) to 11.85 (SD 1.57). The statistical significance of the differences between the PRP and control groups was established (*p* = 0.037 and *p* < 0.001, respectively).	NA

Abbreviations: PRP, Platelet-Rich Plasma; RCT, Randomized Controlled Trial; M, Male; F, Female; COVID-19, Coronavirus Disease 2019.

## Data Availability

All the data reported are present on PubMed web database.
